# Differential Expression of CD96 Surface Molecule Represents CD8^+^ T Cells with Dissimilar Effector Function during HIV-1 Infection

**DOI:** 10.1371/journal.pone.0051696

**Published:** 2012-12-13

**Authors:** Emily M. Eriksson, Chris E. Keh, Steven G. Deeks, Jeffrey N. Martin, Frederick M. Hecht, Douglas F. Nixon

**Affiliations:** 1 Division of Experimental Medicine, Department of Medicine, University of California San Francisco, San Francisco, California, United States of America; 2 HIV/AIDS Program, Department of Medicine, University of California San Francisco, San Francisco, California, United States of America; 3 Department of Epidemiology and Biostatistics, University of California San Francisco, San Francisco, California, United States of America; Massachusetts General Hospital, United States of America

## Abstract

During HIV-1 infection, immune dysregulation and aberrant lymphocyte functions are well-established characteristics. Cell surface molecules are important for immunological functions and changes in expression can affect lymphocyte effector functions, thereby contributing to pathogenesis and disease progression. In this study we have focused on CD96, a member of the IgG superfamily receptors that have generated increasing recent interest due to their adhesive and co-stimulatory functions in addition to immunoregulatory capacity. CD96 is expressed by both T and NK cells. Although the function of CD96 is not completely elucidated, it has been shown to have adhesive functions and enhance cytotoxicity. Interestingly, CD96 may also have inhibitory functions due to its immunoreceptor tyrosine-based inhibitory motif (ITIM). The clinical significance of CD96 is still comparatively limited although it has been associated with chronic Hepatitis B infection and disease progression. CD96 has not previously been studied in the context of HIV-1 infection, but due to its potential importance in immune regulation and relevance to chronic disease, we examined CD96 expression in relation to HIV-1 pathogenesis. In a cross-sectional analysis, we investigated the CD8^+^ T cell expression of CD96 in cohorts of untreated HIV-1 infected adults with high viral loads (non-controllers) and low viral loads (“elite” controllers). We demonstrated that elite controllers have significantly higher CD96 mean fluorescence intensity on CD8^+^ T cells compared to HIV-1 non-controllers and CD96 expression was positively associated with CD4^+^ T cell counts. Functional assessment showed that CD8^+^ T cells lacking CD96 expression represented a population that produced both perforin and IFN-γ following stimulation. Furthermore, CD96 expression on CD8^+^ T cells was decreased in presence of lipopolysaccharide *in vitro*. Overall, these findings indicate that down-regulation of CD96 is an important aspect of HIV-1 pathogenesis and differential expression is related to cell effector functions and HIV-1 disease course.

## Introduction

The rate at which an HIV-1 infected individual progresses to AIDS is dependent on a number of factors, including genetic background and the ability of the immune system to respond to infection. The importance of CD8^+^ T cells during HIV-1 infection has been well-established to play a key role in the control of viremia, where emergence of HIV-1-specific CD8^+^ T cells are associated with rapid decrease of viral load [1], [2], [3], [4], [5], [6], [7], [8]. However, despite the appearance of HIV-1-specific CD8^+^ T cell responses, the majority of HIV-1 infected individuals will eventually develop AIDS. The underlying mechanisms for this are not completely understood, but may potentially be due to impaired immune regulation by CD8^+^ T cells that subsequently influence effector cell functions.

We investigated the effect of HIV-1 infection on the expression of CD96, which is also called *T* cell *ACT*ivating *I*ncreased *L*ate *E*xpression (TACTILE). CD96 was originally identified as a ubiquitously expressed T cell receptor, but can also be found on NK cells [9]. CD96, along with CD226 (DNAM-1), Class-I MHC-restricted T-cell-associated molecule (CRTAM) and T cell immunoreceptor with Ig and ITIM domains (TIGIT), comprise a group of IgG superfamily receptors. All of these molecules share similar structural motifs and bind nectins and nectin-like (Necl) proteins. Initially they were believed to mainly serve as adhesion molecules. However, all members of this group have now been associated with enhancing or influencing lymphocyte functions [10], [11], [12], [13], [14]. CD155, also called poliovirus receptor or Necl-5, is the ligand for CD96, CD226 and TIGIT. CD226 interaction with CD155 is involved in the cytolytic function for both NK cells and T cells [13], [15]. Furthermore, there is a functional link between CD226 and lymphocyte function-associated antigen 1 (LFA-1), where CD226 acts as a LFA-mediated co-stimulatory molecule and have been suggested to be involved in the regulation of T cell activation [11], [16]. More recent studies also show that TIGIT, which has an immunoreceptor tyrosine-based inhibitory motif (ITIM), function as a T cell inhibitor [17]. In contrast to these receptors, CD96 function is not well characterized. Although CD96 also contains an ITIM, interactions between CD96 and CD155 result in enhanced NK cell cytotoxicity [12]. However, the functional role of CD96 on T cells still remains to be determined.

Apart from morphological changes in infected cells, surface receptors with adhesive and immunoregulatory functions, including the IgG superfamily, expressed on bystander cells could also potentially be affected by HIV-1-induced chronic immune activation and inflammatory responses. The consequences of a modified ability to form a stable interaction with a target cell or an antigen-presenting cell, or changes in immune regulation could thereby render cells either incapable of responding optimally to pathogens or provide a mechanism for hyperactivity. Either outcome would have detrimental effects during HIV-1 infection. Considering that chronic disease-associated alteration in CD96 has already been observed during Hepatitis B infection [18], we aimed to investigate changes in expression of CD96 during HIV-1 infection, a receptor with potential importance for effector functions. Our data provide further support that CD96 expression is closely related to chronic infection and disease progression and signify an additional measure of cell function capacity that may prove useful for monitoring of HIV-1 related pathogenesis.

## Materials and Methods

### Study Subjects

Cryopreserved peripheral blood mononuclear cells (PBMCs) from a total of 40 HIV-1-infected subjects from the University of California San Francisco (UCSF) SCOPE cohort were assessed. These study participants were divided into two well-characterized groups (i) “elite controllers” (EC), defined as subjects who maintained undetectable HIV-1 RNA levels (<50–75 copies/ml) for at least two years without ART and a proximal CD4^+^ T cell count of above 350 cells/mm^3^ (n = 20), and (ii) HIV-1 non-controllers (NC), defined as untreated subjects who had HIV-1 RNA levels greater than 2000 copies/ml (n = 20). PBMCs from 30 healthy blood donors from the Stanford Blood Center, Palo Alto, CA and 10 healthy blood donors from San Francisco, CA were included as controls. This study was approved by the UCSF Committee on Human Research and all subjects provided written informed consent to participate in this study, in accordance with the Declaration of Helsinki.

### Flow Cytometry

A total of 5×10^5^ PBMCs was surface stained with antibody mixtures in FACS buffer (phosphate buffer saline containing 0.5% bovine serum albumin (BSA) and 2 mM ethylenediaminetetraacetic acid (EDTA)) on ice for 30 min. Antibodies used included: Alexa700-conjugated anti-CD4 (clone RPA-T4), phycoerythrin-Cy7 (PE-Cy7)-conjugated anti-CCR7 (clone 3D12), PerCP Cy5.5-conjugated anti-HLA-DR (clone L243 (G46-6)), PE-Cy7-conjugated anti-CD38 (clone HB7), Pacific Blue (PB)-conjugated anti-CD3 (clone UCHT1), allophycocyanin-Cy7 (APC-Cy7)-conjugated anti-CD4 (clone SK3), (all from BD Biosciences, San Jose, CA), Qdot 605-conjugated anti-CD8 (clone 3B5; Invitrogen, Carlsbad, CA), PE-Texas Red, (ECD)-conjugated anti-CD28 (clone CD28.2; Beckman Coulter, Brea, CA), Alexa700-conjugated anti-CD45RA (clone HI100; BioLegend, San Diego, CA), Alexa647-conjugated anti-CD226 (clone DX11; BioLegend) and PE-conjugated anti-CD96 (clone NK92.39; eBioscience, San Diego CA). Aqua live/dead amine reactive dye (Invitrogen) was used for dead cell exclusion. Samples were analyzed on a customized four-laser LSR II flow cytometer (BD Biosciences). Data analysis was performed using FlowJo software (TreeStar, Ashland, OR).

### ELISPOT Assay

Functional assessment of FACS sorted cells for both IFN-γ and perforin production was performed following stimulation with phorbol myristate acetate (PMA) (50 ng/ml) in combination with ionomycin (1 µg/ml). ELISPOT plates were coated with either 2.5 µg/ml anti-IFNγ antibody (clone 1D1K, MabTech, Nacka, Sweden) or 30 µg/ml anti-perforin antibody (clone Pf-80/164, MabTech). Following cell stimulation for 16–18 hrs cytokine production was detected with either 1 µg/ml biotinylated anti- IFNγ antibody (clone MabTech) or 2 µg/ml biotinylated anti-perforin (clone Pf-344, MabTech). Spot quantification was achieved with an AID automated ELISpot reader (Cell Technology International, Columbia, MD). FACS sorted cells were assayed in single wells and spot forming units (SFU) were calculated following background subtraction from wells with cells in media only.

### Cell Sorting

PBMCs from healthy individuals were stained with PB-conjugated anti-CD3, Alexa700-conjugated anti-CD4, APC-Cy7-conjugated anti-CD8 and PE-conjugated anti-CD96. CD8^+^ T cells were sort-purified based on CD96 staining using a BD FACS Aria flow cytometer (BD Biosciences). Purity of sorted cell populations was consistently > 96 %.

### CD96 Expression Following Stimulation

PBMCs (5×10^5^ cells) from healthy individuals were stimulated with either lipopolysaccharide (LPS, 1 µg/ml), PHA (1 µg/ml), IL-12 and IL-18 (50 ng/ml of each, Peprotech, Rocky Hill, NJ) or anti-CD3 (clone HIT3α, 1 µg/ml; BD Biosciences) in combination with anti-CD28 (L293, 1 ug/ml; BD Biosciences) for 24 hrs. Cells were surface stained with PE-conjugated anti-CD96, Alexa700-conjugated anti-CD4, APC-Cy7-conjugated anti-CD8. ECD-conjugated anti-CD3 expression was determined following cell permeabilization with FACS permeabilizing solution 2 (BD Biosciences) and intracellular staining.

### Statistical Analysis

All statistical analyses were performed using Prism 4.0 (GraphPad software). Flow cytometry data was analyzed using either Kruskal-Wallis test followed by the Dunn post-test or a Student’s T test as indicated. Correlation coefficients were determined by Spearman rank correlation. *P* values were based on two-tailed tests and results < 0.05 were considered statistically significant.

## Results

### CD96 is Down-regulated on CD8^+^ T Cells in HIV-1 Non-controllers

**Figure 1:**
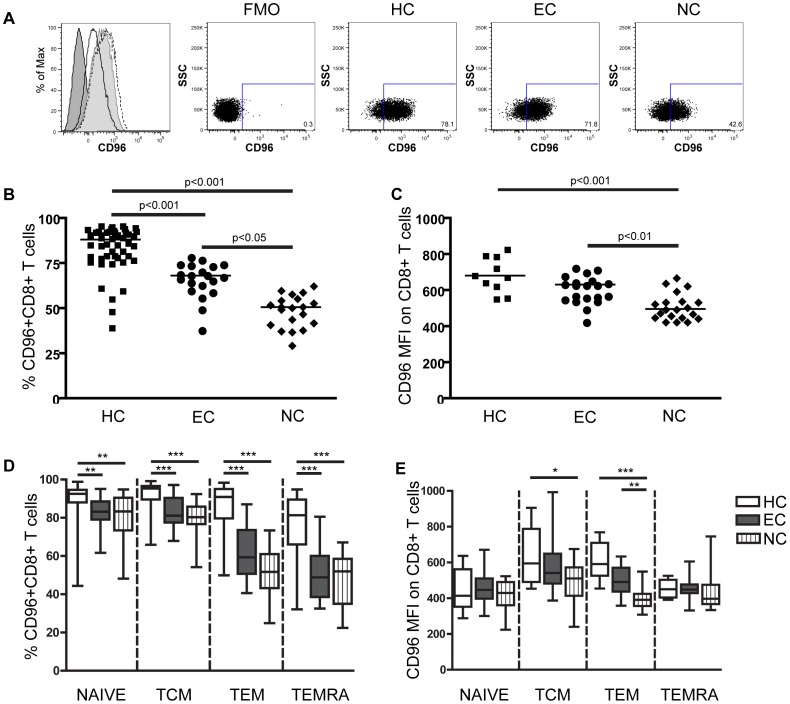
CD96 T cell expression in HIV-1-infected subjects is down-regulated compared to healthy controls (HC). PBMCs from elite controllers (EC, n = 20), viremic non-controllers (NC, n = 20) and healthy controls (HC, n = 40) were surface stained for CD96 expression. A) Representative histograms (dark grey = fluorescence minus one (FMO) control, solid black line = NC, light grey = EC, dotted black line = HC) and dot plots. B) Percentage of CD8^+^ T cells expressing CD96. C) Mean fluorescence intensity (MFI) of CD96 on CD8^+^ T cells. D) Percentage of naïve and different CD8^+^ T cell memory populations (TCM = central memory T cell, TEM = effector memory T cell, TEMRA = terminally differentiated effector memory T cell) expressing CD96. E) CD96 MFI on CD8^+^ T cells within each memory subset. The bars represent the mean and error bars represent the range from minimum to maximum value. Statistical analysis was performed using Kruskal Wallis tests with Dunn’s post-test *p < 0.05, **p < 0.01, ***p < 0.001.

The expression of CD96 during HIV-1 infection has not previously been assessed, but based on reports that CD96 is up-regulated during T cell activation [9] we hypothesized that CD96 would be higher in HIV-1 patients due to persistent immune activation. To determine if the CD96 expression was changed during HIV-1 infection, we assessed CD96 expression by CD8^+^ T cells in elite controllers (EC) (mean 961 CD4^+^ T cells/mm^3^ and < 50 RNA copies/ml), HIV-1 non-controllers (NC) (mean 536 CD4^+^ T cells/mm^3^ and mean 68,049 RNA copies/ml) and healthy controls (HC). Representative histograms and dot plots of CD96 expression in healthy controls (HC), elite controllers (EC) and non-controllers (NC) are shown in Figure 1A, where CD96 expression was determined based on fluorescence minus one (FMO) control. We found that a high percentage of resting CD8^+^ T cells expressed CD96 in healthy individuals (Fig. 1B). Unexpectedly, the frequency of CD96-expressing CD8^+^ T cells was significantly lower in both HIV-1 infected groups (p < 0.001 for both groups) compared to healthy controls (HC) (Fig. 1B). However, the frequency of CD96-expressing CD8^+^ T cells was significantly higher in the EC group compared to the NC group (p < 0.05). Furthermore, the CD96 mean fluorescence intensity (MFI) on CD8^+^ T cells was significantly lower in the NC group (mean MFI = 510) compared to the healthy controls (mean MFI = 690, Fig. 1C). In contrast the mean CD96 MFI in the EC group (mean MFI = 606) was comparable to healthy controls and was significantly higher than that of the NC group (p < 0.01). To determine if the lower frequencies in CD96-expressing CD8^+^ T cells were a reflection of changes in the total T cell composition commonly seen in HIV-1 infected individuals, we also assessed absolute numbers of CD96^+^ T cells. We found that the number of CD8^+^ T cells expressing CD96 was similar in both HIV-1 groups and healthy controls suggesting that the total CD8^+^ T cell subset population was expanded in HIV-1 infected patients, with an increased CD96 negative population (data not shown). In contrast to CD96 expression and a previous report [19] CD226 expression on CD8^+^ T cells was unchanged in all HIV-1 infected subjects (data not shown). All together these results show that even though CD96 is considered a T cell activation marker, HIV-1 infection, in particular uncontrolled infection, appears to promote down-regulation of CD96 expression. Furthermore, despite the close relationship and similar characteristics of CD96 and CD226, they are differentially affected by HIV-1 infection.

### CD96 is not Lost Due to CD8^+^ T Cell Differentiation

HIV-1 infection promotes a higher degree of CD8^+^ T cell differentiation and the total population of naïve cells in the periphery is diminished. To establish if CD96 expression was down-regulated following differentiation and thus account for the lower frequencies of CD96-expressing CD8^+^ T cells observed in the HIV-1 infected individuals of this study, we assessed CD96 expression on CD8^+^ T cell subsets. The CD8^+^ T cell population was divided into T cell subsets determined by CD45RA and CCR7 expression. Cell populations were defined as CCR7^+^CD45RA^+^ naïve cells, CCR7^+^CD45RA^neg^ central memory T cells (TCM), CCR7^neg^CD45RA^neg^ effector memory T cells (TEM) and CCR7^neg^CD45RA^+^ terminally differentiated effector memory T cells (TEMRA). The frequency of CD96-expressing cells in all subsets was significantly lower in both EC and NC (Fig. 1D, Naïve subset p<0.01 and memory subsets p<0.001 for both groups). Although, the frequencies of CD96-expressing naïve and TCM CD8^+^ T cells were significantly lower than in healthy controls, the majority of cells in these subsets maintained CD96 expression. The CD96 MFI in all subsets was comparable between EC and healthy controls (Fig. 1E). In the NC group, the CD96 MFI was slightly reduced in the TCM cell subset compared to healthy controls (p < 0.05). Additionally the CD96 MFI in the TEM cell subset was significantly lower in the NC group compared to both healthy controls (p < 0.001) and the EC group (p < 0.01). Since we had observed that the CD96 MFI for the total CD8^+^ T cell population was higher in the EC group than in the NC group (Fig. 1C), this suggests that the difference in CD96 MFI between these groups was predominantly attributed to differential CD96 expression within the TEM population. Collectively, these results also indicate that CD8^+^ T cell differentiation alone is not sufficient to down-regulate CD96 expression.

### CD96 is Down-regulated Following Stimulation with LPS

**Figure 2:**
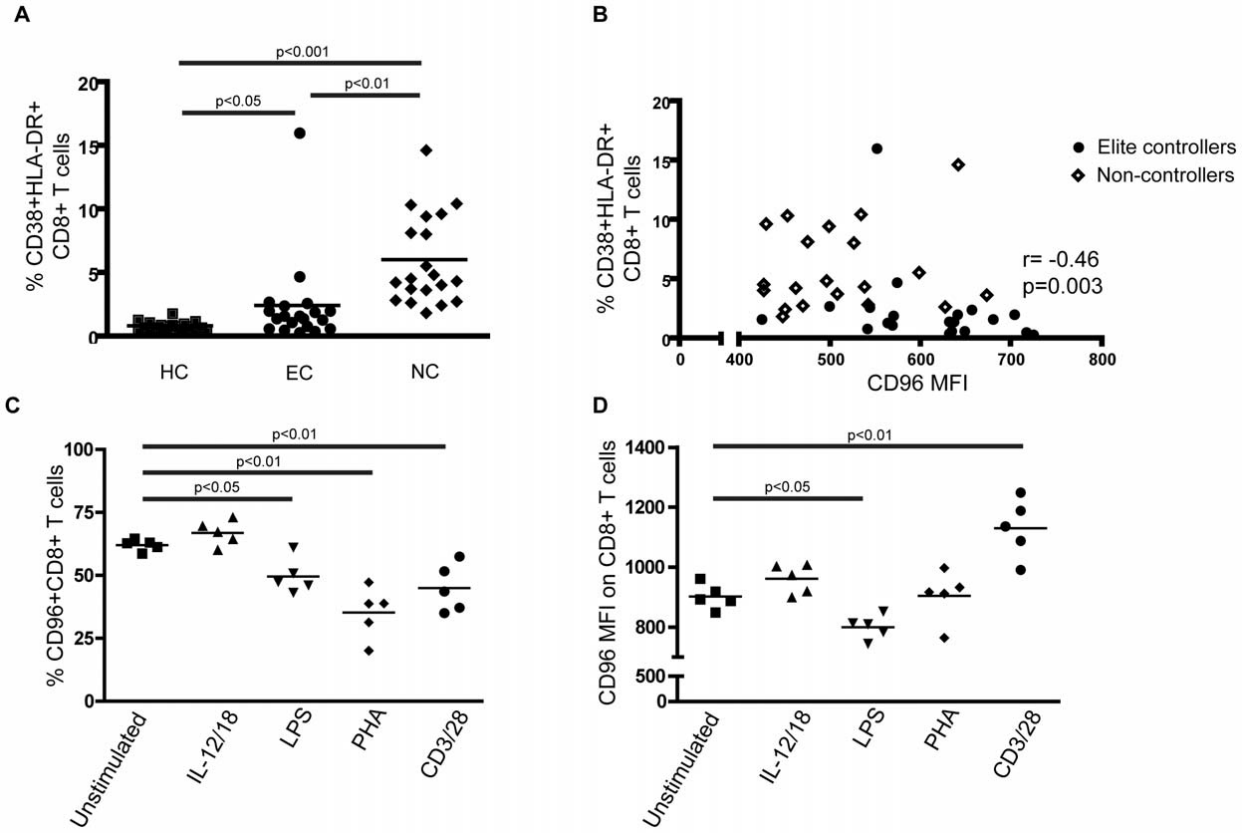
CD96 expression is down-modulated by LPS stimulation and up-regulated by TCR engagement. A) Percentage of CD38^+^ HLA-DR^+^ CD8^+^ T cells as a measure of immune activation. B) Association of CD96 MFI on CD8^+^ T cells and percentage of CD38^+^ HLA-DR^+^ CD8^+^ T cells (n = 40). C) Percentage of CD96 expression and D) CD96 MFI on CD8^+^ T cell following stimulation with either LPS, PHA, IL-12/18 and anti-CD3/CD28 for 24 hrs compared to unstimulated cells. Statistical analysis was performed using Student’s T test *p < 0.05, **p < 0.01, ***p < 0.001. Correlations were determined by two-tailed non-parametric Spearman correlations.

Chronic immune activation is a well-established characteristic of HIV-1 infection and has been associated with factors such as inflammatory responses, the presence of LPS in plasma as a consequence of microbial translocation [20] and low levels of virus replication [21], [22] resulting in continuous antigen stimulation of T cells. We assessed the immune activation status of individuals in our cohort measured by presence of CD38 and HLA-DR double-positive CD8^+^ T cells and observed that immune activation was significantly higher in both the EC group (p < 0.05) and NC group (p < 0.001) compared to healthy individuals (Fig. 2A). However, the EC group had significantly lower immune activation compared to the NC group (p < 0.01). Furthermore, we found that immune activation was negatively correlated to both frequency of CD96^+^ CD8^+^ T cells (data not shown) and CD96 MFI (Fig. 2B, r = -0.46 p = 0.003, n = 40). However, there was no correlation between immune activation and CD96 MFI for the NC group alone. To determine if some of the stimuli related to immune activation may influence CD96 expression on CD8^+^ T cells, we stimulated PBMCs from healthy individuals *in vitro* with LPS, IL-12/IL-18, PHA or CD3 in combination with CD28. The frequency of CD96^+^CD8^+^ T cells was significantly lower after LPS (mean = 49.7%), PHA (mean = 35.3 %) and anti-CD3/28 (mean = 45%) stimulation compared to unstimulated cells (mean = 62.1 %, Fig. 2C). Similarly, the CD96 MFI was significantly lower on CD8^+^ T cells following LPS stimulation compared to unstimulated cells (mean MFI 800.2 vs 901.8, Fig. 2D). In contrast stimulation with anti-CD3/28 increased the CD96 MFI (mean MFI = 1130) whereas CD96 MFI was maintained following stimulation with PHA. There was no significant difference in either percentage or CD96 MFI following IL12/18 stimulation. This suggests that differential stimulation had distinctive effects on CD96 expression.

### CD8^+^ T Cells Lacking Expression of CD96 Produce Both IFN-γ and Perforin

**Figure 3:**
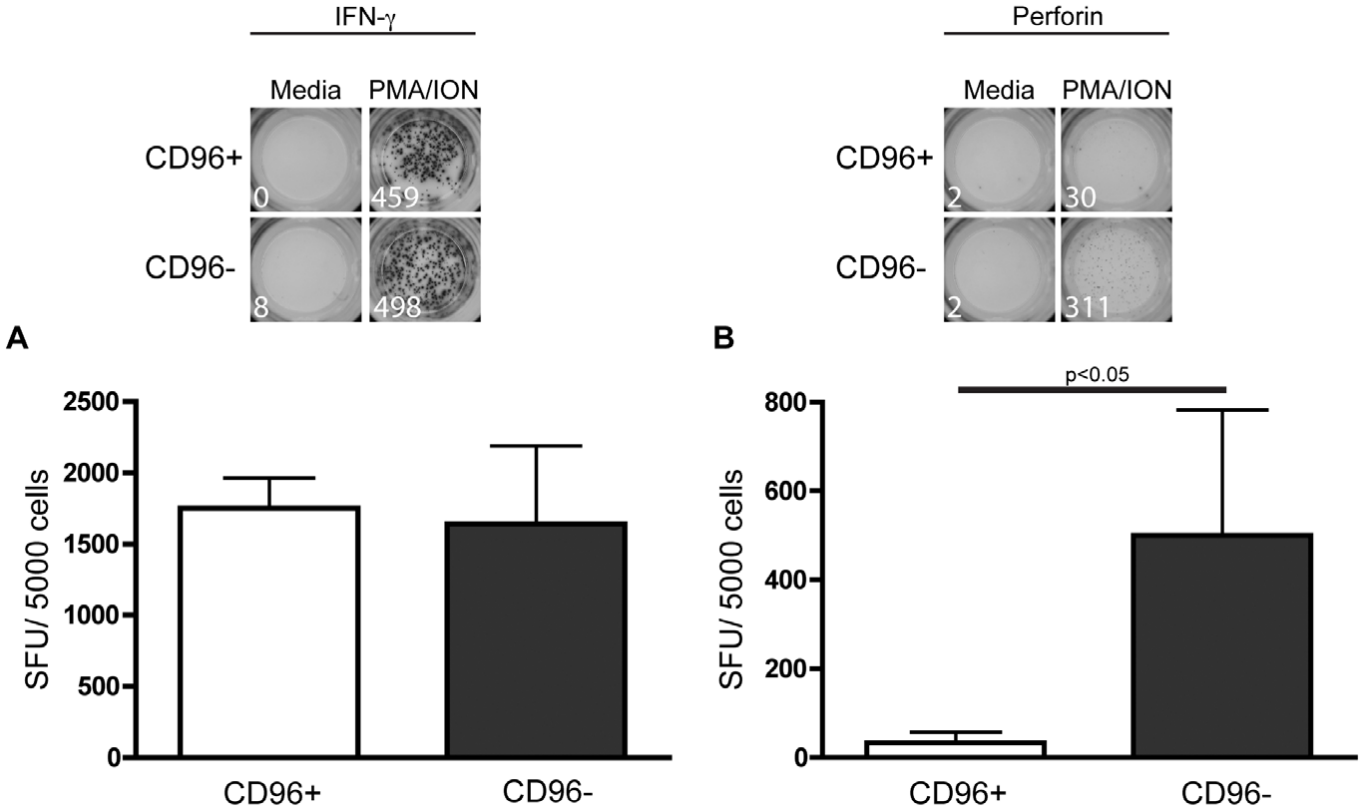
CD8^+^ T cells lacking CD96 produce perforin and IFNγ following stimulation with PMA/ionomycin. FACS sorted CD96^+^ and CD96^neg^ were stimulated with PMA/ionomycin and assessed for A) IFNγ and B) perforin production in an ELISPOT assay. Bars represent the mean value ± SD of three independent experiments and show spot forming units (SFU) per 5000 cells. Statistical analysis was performed using Student’s T test *p < 0.05.

We have established that the frequency of CD96 expression is modified during HIV-1 infection and that the density of CD96 per cell is decreased in non-controllers suggesting that cells lacking CD96 may potentially be dysfunctional. Consequently, we investigated functional differences between CD96-expressing CD8^+^ T cells and CD8^+^ T cells lacking CD96 from healthy individuals. As we found that differential stimulation could modulate CD96 expression, we sorted cells based on their CD96 expression prior to stimulation. We found that *both* CD96^+^ and CD96^neg^ CD8^+^ T cells produced high levels of IFN-γ following PMA/ionomycin stimulation (Fig. 3A). Interestingly, the CD8^+^ T cells lacking CD96 surface expression also produced perforin (Fig. 3B) demonstrating that these cells were not functionally impaired, but were instead highly cytotoxic.

### Decreased CD96 MFI and Absolute Numbers is Associated with Disease Progression

**Figure 4:**
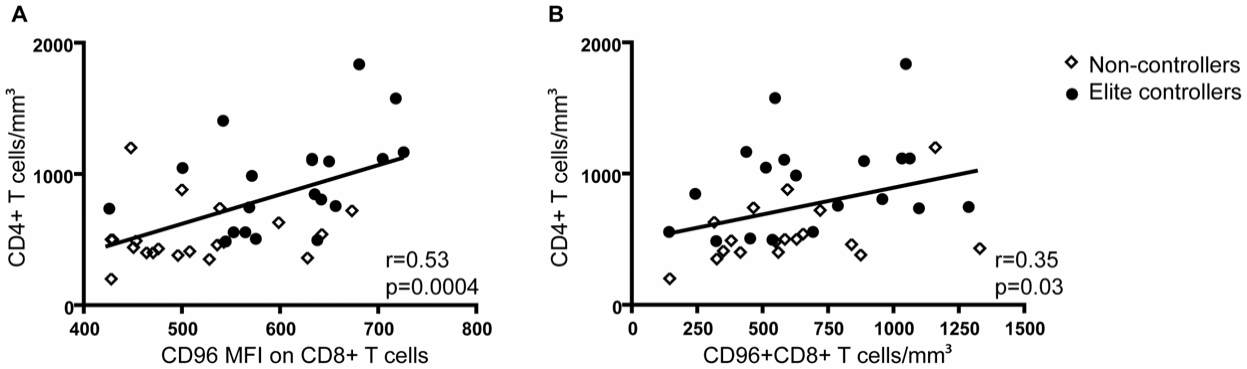
The absolute number and CD96 MFI of CD96^+^CD8^+^ T cells correlates with CD4^+^ T cell counts. Association of A) CD96 MFI on CD8^+^ T cells (n = 37) and B) the number of CD96^+^CD8^+^ T cells with CD4^+^ T cell counts (n = 36). Correlations were determined by two-tailed non-parametric Spearman correlations.

One of the hallmarks of HIV-1 disease progression is CD4^+^ T cell depletion. We have shown that during HIV-1 infection the CD8^+^ T cell population express lower levels of CD96. These cells produce perforin following stimulation *in vitro*. Presence of abnormally high fractions of cytotoxic T cells could potentially contribute to immunopathogenesis and increased destruction of CD4^+^ T cells. Thus, to determine a potential clinical relevance of CD96, we assessed CD96 expression relative to CD4^+^ T cell counts. Due to the relatively small sample size of each group, correlations were assessed on the total number of HIV-1 infected individuals. We found that both the CD96 MFI on CD8^+^ T cells (r = 0.53 and p < 0.0004, n = 37) and the absolute number of CD96^+^CD8^+^ T cells (r = 0.35 and p = 0.03, n = 36) were positively correlated with CD4^+^ T cell counts (Fig. 4A and B). Commonly, high viral loads can be associated with lower CD4^+^ T cell counts. However, in the viremic individuals included in this study cohort no association was observed (data not shown). Instead, we established that the absolute numbers of TEM cells was positively correlated with viral load in viremic patients regardless of CD96 expression (data not shown). Collectively this suggested that disease progression, as determined by CD4^+^ T cell counts, was correlated with CD96 expression, but was unaffected by viral loads. Instead the presence of virus promoted the expansion of the CD8^+^ TEM cell pool, but was not directly associated with CD96 expression.

## Discussion

CD96 is normally expressed by most T cells. However, the function of CD96 expression on T cells still remains elusive. Furthermore, modulations in CD96 expression during disease such as HIV-1 infection and relationship to pathogenesis has not previously been reported. In this study, we have shown that elite controllers significantly differ in their expression of CD96 as compared to non-controllers. The reduced frequency of CD96 expressing CD8^+^ T cells were observed in all T cell subsets, although decreased density of CD96 expression was predominantly observed in the TEM population. The absolute numbers of CD96^+^ CD8^+^ T cells and the MFI were significantly associated with the peripheral CD4^+^ T cell counts. Collectively these data suggest that CD96 is potentially causally related to prevention of HIV-1-associated disease progression, although the cross-sectional nature of the study precludes definitive conclusions. Furthermore, we found that presence of LPS (which is thought to drive pathogenesis in HIV-1 disease) promoted CD96 down-regulation *in vitro* whereas direct TCR stimulation with anti-CD3 and anti-CD28, but not PHA, resulted in increased per cell CD96 density. We also observed that cells lacking CD96 on CD8^+^ T cells represented a population that produced both IFNγ and perforin following stimulation. All together, these data suggest that changes in CD96 expression may be a useful additional marker to measure overall effector function and disease progression during HIV-1 infection.

In HIV-1 infected individuals chronic immune activation is a common feature where increased CD8^+^ T cell activation is associated with CD4^+^ T cell depletion [23], [24]. During HIV-1 infection there is also evidence that bacterial translocation occurs as LPS and bacterial DNA have been detected in the blood of HIV-1 infected individuals [20], [25]. CD96 is abundantly expressed on resting T cells, but interestingly we found that *in vitro* LPS stimulation of PBMCs from healthy donors decreased CD96 expression. It has already been reported that CD96 can be shed during chronic disease such as Hepatitis B infection [18]. Correspondingly, we observed that CD96 expression was decreased during chronic HIV-1 infection in our cohort. The total CD8^+^ T cell population of elite controllers was also found to have decreased frequencies of CD96^+^ expressing cells compared to healthy controls, although density per cell was maintained. These data indicate that inflammatory responses to LPS is a contributing mechanism by which down-regulation of CD96 expression is induced. LPS translocation may therefore to some extent explain the down-regulation of CD96 observed in the subjects of this cohort. In contrast to LPS, direct TCR stimulation *in vitro* instead increased the density of CD96 expression. This is in accordance with previous studies that report upregulation of CD96 on T cells upon activation [9]. However, TCR triggering by PHA resulted in unchanged CD96 MFI. Furthermore, *in vitro* TCR stimulation consistently demonstrated decreased percentage of CD96-expressing CD8^+^ T cells. This suggests that the context in which TCR engagement is conducted contributes to the outcome in CD96 regulation and it is possible that a balance of both LPS and antigenic stimulation affect final CD96 expression and composition in the T cell population *in vivo* of HIV-1 infected individuals.

CD96 has been shown to enhance NK cell cytotoxicity [12], where the specific function on T cells has not been completely defined. We hypothesized that CD96 may play a similar role for CD8^+^ T cell cytotoxic capacity. Since cytotoxic ability is increased with CD8^+^ T cell differentiation, we expected that CD96 would predominantly be present on more differentiated cells. However, we found that CD96 was expressed in all CD8^+^ T cell subset populations. When investigating the significance of CD96 expression by CD8^+^ T cells, we observed that cells which retained CD96 expression produced IFNγ following stimulation. In contrast, cells lacking CD96 readily produced *both* perforin and IFNγ suggesting that these cells were highly active and cytotoxic cells. It is possible that circulating CD96^neg^ CD8^+^ T cells represent a subset of effector memory cells not distinguishable using our surface markers, which are known to produce both perforin and cytokine [26].

Perforin is an effector molecule necessary for cytotoxic activity, which mediates destruction of virus-infected cells. As expected, perforin has been found to be a marker of viral control in HIV-1 infected individuals where elite controllers have previously been shown to have a higher degree of perforin-expressing HIV-1-specific CD8^+^ T cells [6], [7], [27], [28], [29]. However, misdirected or overproduction of perforin in an HIV-1 infected individual could potentially result in increased immunopathogenesis and a drop in CD4^+^ T cell counts. In support of this is the observation that elevated perforin levels were detected in serum from chronically infected individuals [30]. Similar to Onlamoon et al. (2012) [31] we investigated the bulk CD8^+^ T cell population in untreated HIV-1 infected individuals rather than HIV-1-specific CD8^+^ T cells to get a better understanding of the general effector status that potentially contributes to overall immunopathogenesis. Consistent with the reported finding of a generalized altered functional CD8^+^ T cell phenotype during HIV-1 infection [31], we found an increase in CD96^neg^ CD8^+^ T cells in HIV-1 infected individuals, which based on our observations in healthy individuals represent highly active and cytotoxic cells producing both IFNγ and perforin. Furthermore, the density of CD96 expression as well as presence of CD96^+^ CD8^+^ T cells were positively associated with CD4^+^ T cell counts. Although, the function of CD96^neg^ CD8^+^ T cells was only assessed in healthy individuals and the function of phenotypically equivalent T cells present in HIV-1 infected individuals remain to be confirmed, these observations suggest that there may be a complex balance between beneficial and detrimental outcomes associated with perforin levels during HIV-1 infection. More specifically, it is possible that HIV-1-specific T cells are required to produce perforin in order to control virus whereas overproduction or HIV-1 non-specific perforin production is characteristic of disease progression.

In conclusion, our results demonstrate a close relationship between CD96 and HIV-1 disease progression and pathogenesis. It is clear that the effect of HIV-1 related inflammatory responses and chronic immune activation have an impact on selected molecules, which indirectly contribute to the immunopathogenesis. Greater understanding of molecules with implications for effector functions, such as CD96, could provide valuable directions and guidelines in monitoring of HIV-1 related pathogenesis.
